# Peruvian university students’ mental health in crisis: assessing anxiety, depression, fear, and stress during the Russia-Ukraine conflict

**DOI:** 10.3389/fpubh.2025.1522132

**Published:** 2025-03-27

**Authors:** Jeel Moya-Salazar, Eliane A. Goicochea-Palomino, Víctor Rojas-Zumaran, María Jesús Moya-Salazar, Gloría Cruz-Gonzales, Hans Contreras-Pulache

**Affiliations:** ^1^Qualitative Unit, Nesh Hubbs, Lima, Peru; ^2^New-Anatomy Lab, Universidad Norbert Wiener, Lima, Peru; ^3^Faculties of Health Science, Universidad Tecnológica del Perú, Lima, Peru; ^4^Department of Pathology, Hospital Nacional Docente Madre Niño San Bartolomé, Lima, Peru; ^5^Faculty of Medical Technologist, Universidad Nacional Federico Villareal, Lima, Peru

**Keywords:** war, mental health, university students, Russia-Ukraine conflict, depression, fear, stress, anxiety

## Abstract

**Background:**

The conflict between Russia and Ukraine, exacerbated by the COVID-19 pandemic, has created global fears over the possibility of another third world war. Populations both close to and far from the conflict can be affected by it and the mental health outcomes can be devastating. We aimed to assess levels of anxiety, depression, fear, and stress among Peruvian university students facing potential war-related consequences.

**Methods:**

This web-based observational study conducted in 2022 included 494 randomly selected students. Participants were over 18 years of age, with a balanced representation of gender and geography. Anxiety, depression, stress, and fear of war were evaluated using validated instruments, including the Generalized Anxiety Disorder-7 (GAD-7) and the Patient Health Questionnaire-9 (PHQ-9).

**Results:**

Of the participants, 58.3% were women, with an average age of 26.4 ± 8.1 years. Symptoms of stress, fear, anxiety, and depression affected 49.2, 49, 47.8, and 47.8%, respectively. Women were more affected, with 54.2% reporting high stress and 53.5% reporting high fear (*p* < 0.001). The most vulnerable group for anxiety and depression were younger students aged 18–20, with 44.3% presenting mild anxiety and 44.3% mild depression (*p* < 0.001).

**Conclusion:**

These findings highlight the urgent need for mental health interventions for university students, particularly women and younger students, who face heightened vulnerability to global crises such as wars and pandemics. Further studies should explore additional influencing factors, including pre-existing health conditions, academic challenges, and regional disparities, to address these mental health issues better.

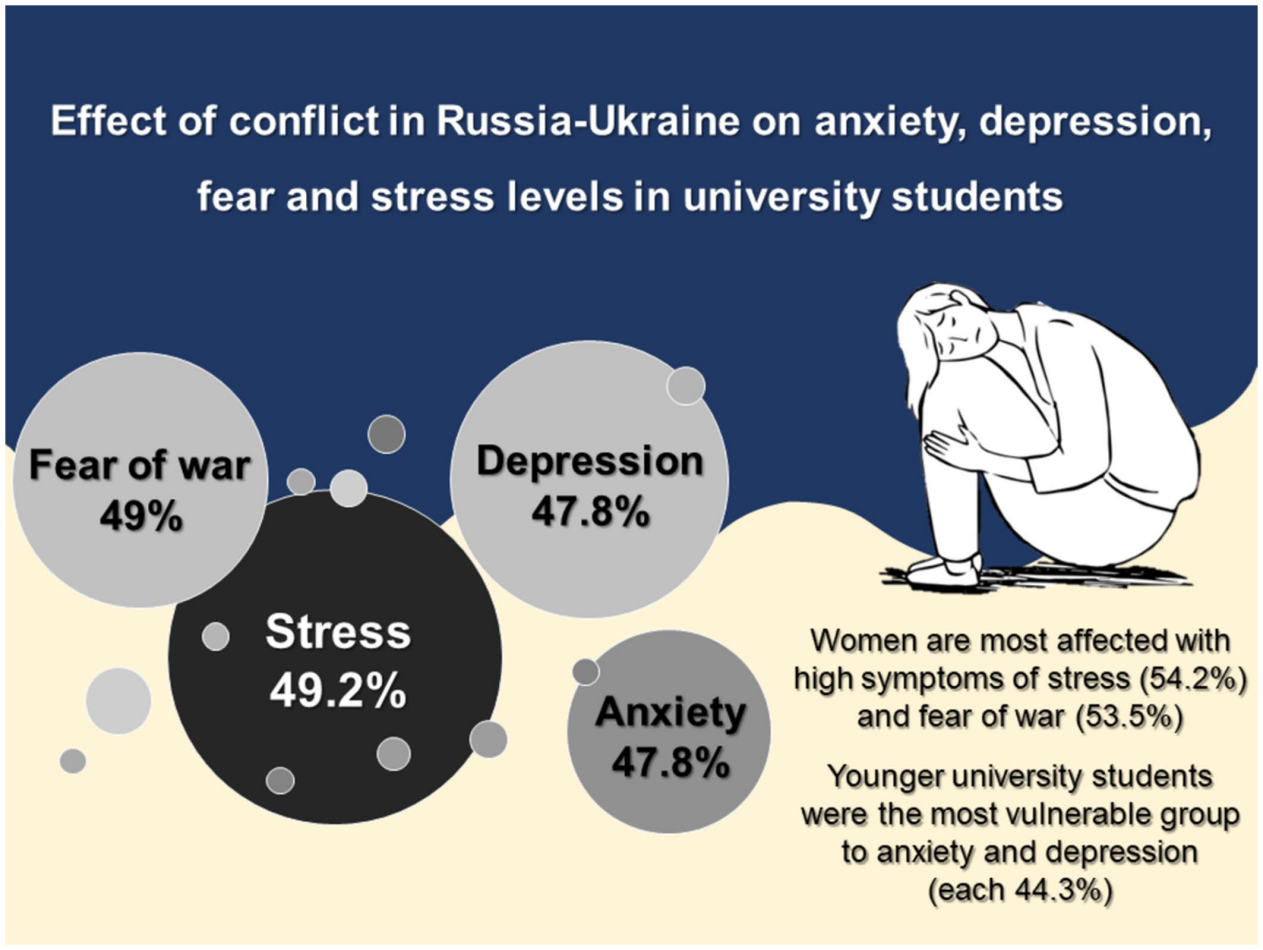

## 1 Introduction

Throughout history, armed conflicts have led to devastating consequences for both societies and individuals, leaving profound physical and psychological scars (1). These extreme scenarios of violence expose populations to traumatic experiences that can endure for decades. For instance, during World War I, an estimated 22 million people died, approximately 13 millions of whom were civilians. These deaths were due to military combat and the 1918 influenza pandemic, known as the “Great Flu,” the deadliest influenza outbreak recorded (2).

Beyond the loss of life, wars also leave deep psychological scars. Soldiers in World War I experienced what was then termed “war neurosis,” characterized by nervous breakdowns during combat, a condition severely underestimated at the time (3). Similarly, during the Nepal conflict (1996–2006), individuals exposed to multiple traumatic events showed a significant increase in fear levels, rising 1.45 times for each additional traumatic experience (4). Another study conducted during the Gaza conflicts found that adolescents who had lived through three consecutive wars reported up to five nightmares per week, many related to attacks and death threats. This led to sleep disturbances, daytime fatigue, and impaired academic performance (5).

Wars not only affect people during conflict but also exacerbate mental health challenges during overlapping crises, such as pandemics (2). The arrival of the COVID-19 pandemic triggered a global health emergency, generating hopes in some quarters for a reduction in violence. The United Nations even issued a global ceasefire call. Nevertheless, the Uppsala Conflict Data Program (UCDP) reported more than 80,000 deaths due to armed conflict in 2020 (6). While soldiers and residents of war zones are most at risk of developing disorders such as anxiety, stress, and depression, the impact of conflicts extends to those indirectly involved as well (7).

The armed conflict between Ukraine and Russia, which began in 2014, continued throughout the COVID-19 pandemic, further aggravating the socioeconomic situation and mental health of the population (8, 9). The war has had a global economic impact, as both countries are major agricultural exporters, endangering food security in countries dependent on these exports (10). Additionally, the conflict has caused severe environmental damage through air, water, and soil contamination due to bombings and the destruction of critical infrastructure. The most alarming risk has been the proximity of hostilities to nuclear facilities, heightening fears of radioactive leaks and further exacerbating the psychological distress of the affected population (11).

Due to the ongoing conflict, Ukraine was unable to effectively implement COVID-19 prevention measures, such as social distancing, isolation, and quarantine, significantly increasing the risk of severe complications and mortality (12). Russian invasions disrupted vaccination campaigns and reduced epidemiological data collection just as Ukraine was experiencing its peak infection rates (13). Furthermore, the Ukrainian population faced limited access to healthcare services and supplies, making them even more vulnerable to other threats, such as the poliomyelitis outbreak detected in October 2021, a consequence of low immunization rates (8). Studies suggest that two-thirds of displaced Ukrainian children and adolescents relocated to temporary groups have the potential to spread diseases as they migrate to European countries (14).

The health impacts of the conflict extend beyond Europe. In Latin America, there is a strong correlation between the fear of a potential global-scale armed conflict and severe levels of stress, anxiety, and depression, particularly in societies already facing persistent political and economic insecurity (15). University students have been among the groups most affected by conflict-related anxiety. A study conducted on Czech university students found that 22.3% of respondents experienced moderate anxiety (while 13.7% suffered from severe anxiety), as well as 22% showed moderate depression, 11% moderately severe, and 7.1% severe depression (16).

These figures highlight the pressing need for further research to better understand both the immediate and long-term psychological impacts of conflict, as well as its indirect effects (17). Such studies are crucial for developing targeted interventions aimed at mitigating the mental health consequences associated with these crises. Additionally, the conflict has generated widespread anger among affected populations, leading to recommendations for implementing emotional regulation strategies. These strategies are vital to prevent psychological distress from escalating into aggressive behavior or policymaking. Instead, the focus should be on fostering humanitarian support and promoting the adoption of conciliatory policies (18).

During the COVID-19 pandemic in Peru, widespread fear of infection was prevalent among the adult population, significantly contributing to sleep disorders (19, 20). This fear was further amplified by poor information dissemination through traditional media, which increased anxiety levels. Exaggerated and misleading information circulating on social media exacerbated these effects. Individuals with only a high school education were particularly affected, with men reporting higher levels of generalized fear (21). The psychological distress caused by the pandemic could be further compounded by the ongoing Russia-Ukraine conflict, potentially worsening the population’s overall quality of life (22).

We aimed to assess the levels of anxiety, depression, fear of war, and stress among university students in response to the potential consequences of the Russia-Ukraine conflict during the COVID-19 pandemic. We sought to understand the mental health repercussions caused by the constant tension surrounding the potential outbreak of war, and how this may affect not only the daily performance of students but also exacerbate the economic, labor, and social conditions in war-affected countries, creating collateral damage in other nations as well.

## 2 Methods

### 2.1 Study design and settings

This was a web-based observational study conducted on a population of Peruvian university students in 2022. According to data from the Ministry of Education (MINEDU), by the end of 2021, the total number of university students in Peru reached 1,423,731, with 25.5% attending public universities and 74.5% enrolled in private institutions (23). Furthermore, according to the National Institute of Statistics and Informatics (INEI), in the fourth quarter of 2022, the university student population in Metropolitan Lima increased by 36.2%, reaching approximately 389,000 students (24).

### 2.2 Population and inclusion criteria instruments

The study population was selected through random sampling and included 494 university students considering a power of 80%. Inclusion criteria were voluntary participation by adults aged 18 years or older of both sexes, residing in Lima or other provinces of Peru, and with nationality (Table 1). Two instruments created and validated by the authors were used to evaluate stress and fear of war related to the Russia-Ukraine conflict. These instruments comprised 10 and 16 items, respectively. The median score was calculated as a cutoff point to differentiate participants with high and low levels of stress (Median: 28 points) and fear (Median: 51 points). Both questionnaires were validated by a Universidad Norbert Wiener team (data no show), the internal consistency of the fear and war questionnaire was *α* = 0.922 and *α* = 0.911, respectively.

**Table 1:** Baseline sociodemographic characteristics of university students.

Variables	Categories	N	%
Sex	Male	201	40.7
	Female	288	58.3
	Nonbinary	5	1.0
Age group (years)	18–20	106	21.5
	21–30	294	59.5
	31–40	57	11.5
	41–50	24	4.9
	51–60	13	2.6
Marital status	Single	414	83.8
	Married	39	7.9
	Divorced	5	1.0
	Cohabiting relationships	36	7.3
Religion	Catholic	330	66.8
	Christianity	69	14.0
	Atheist	34	6.9
	Agnostic	39	7.9
	Others	22	4.5
City	Lima	459	92.9
	Province	35	7.1
Total		494	100

Additionally, the Generalized Anxiety Disorder-7 (GAD-7), developed by Spitzer et al. in 2006 (25), was used. This tool is based on the core criteria from the Diagnostic and Statistical Manual of Mental Disorders, Fifth Edition (DSM-5), to identify probable cases of Generalized Anxiety Disorder. The GAD-7 was adapted to Spanish by García Campayo et al. in 2010 (26). It consists of a Likert-like 7 items questionnaire, based on symptom frequency over the past 2 weeks: “not at all” (0 points), “several days” (1 point), “more than half the days” (2 points), and “nearly every day” (3 points). According to the total score, anxiety is classified as follows: 0–4 points (no anxiety), 5–9 points (mild anxiety), 10–14 points (moderate anxiety), and 15–21 points (severe anxiety) (27).

The Patient Health Questionnaire-9 (PHQ-9), created by Kroenke et al. in 2001 (28), was used to provide a provisional diagnosis of depression and indicate its severity. This instrument is also based on DSM-5 criteria and was validated for the Peruvian population by Calderón et al. in 2012 (29). The PHQ-9 consists of 9 items, scored on a Likert scale from 0 (not at all) to 3 (nearly every day). Depression severity is classified into five categories: none (0–4 points), mild (5–9 points), moderate (10–14 points), Moderately severe depression (15–19 points), and severe (20–27 points) (30).

### 2.3 Data gathering and analysis

The online survey was conducted in July 2022 via Google Forms (Google, CA), following the recommendations of the Based Checklist for Reporting of Survey Studies (CROSS) (31). Two independent reviews of the participant responses were performed using MS Excel, and any entries with filling errors were excluded during the quality control process. Descriptive statistics were used for the initial data analysis, calculating absolute frequencies, means, and standard deviations (SD) for each questionnaire according to its specific interpretation. Non-paired t-tests and one-way ANOVA with *post-hoc* Bonferroni tests were conducted to identify differences in mental health outcomes (anxiety, depression, fear, and stress) based on demographic characteristics with a *p*-value threshold of 0.05 and a 95% confidence interval considered statistically significant.

### 2.4 Ethical aspects

This study adhered to the guidelines of the Declaration of Helsinki (32) and was approved by the Ethics and Research Committee of Universidad Norbert Wiener (Register No. 916–2022). This study included virtual informed consent, using a standardized code to ensure the anonymity of the participants in compliance with Law 29,733 (33).

## 3 Results

**Figure 1:**
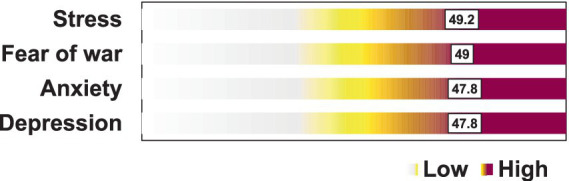
Russia-Ukraine conflict-related mental health issues in university students.

Among the 494 participants, more than half were women (288/494, 58.3%), with only 1% (5/494) identifying as non-binary. The average age was 26.4 ± 8.1 years, with the most common age group being 21–30 (294/494, 59.5%). Most participants were single (414/494, 83.8%), Catholic (414/494, 83.8%), and resided in Lima, the capital of Peru (459/494, 92.9%). Symptoms of stress, fear, anxiety, and depression affected 49.2, 49, 47.8, and 47.8% of university students, respectively (Figure 1).

### 3.1 Stress

Stress affected 49.2% (243/494) of participants, with higher prevalence among women (58.3%, 156/288, *p* < 0.001). We found significant differences in stress symptoms between women and men (*p* = 0.003), but no significant differences with non-binary students (*p* = 0.074). Stress levels varied by age, with the highest prevalence observed among participants aged 51–60 years (61.5%), followed by 18–20 years (51.9%) and 21–30 years (50.3%). Regarding marital status, high stress was more common among divorced participants (80%), singles (50%), and those in cohabiting relationships (47.2%; *p* = 0.072). In terms of religious affiliation, elevated stress was reported by 59.0% of agnostics and 52.4% of Catholics. Geographically, high stress affected 50.3% of students in Lima, compared to 34.3% of those from other provinces (*p* = 0.002; Table 2).

**Table 2:** Distribution of stress symptoms in Peruvian university students.

Variables	Categories	Low stress	High stress	Total	*p*-value
Sex	Male	117(58.2)	84 (41.8)	201 (40.7)	<0.001
	Female	132 (45.8)	156 (54.2)	288 (58.3)	
	No binary	2 (40)	3 (60)	5 (1.0)	
Age group (years)	18–20	51 (48.1)	55 (51.9)	106 (21.5)	0.085
	21–30	146 (49.7)	148 (50.3)	294 (59.5)	
	31–40	29 (50.9)	28 (49.1)	57 (11.5)	
	41–50	20 (83.3)	4 (16.7)	24 (4.9)	
	51–60	5 (38.5)	8 (61.5)	13 (2.6)	
Marital status	Single	207 (50)	207 (50)	414 (83.8)	0.072
	Married	24 (61.5)	15 (38.5)	39 (7.9)	
	Divorced	1 (20)	4 (80)	5 (1.0)	
	Cohabiting relationships	19 (52.8)	17 (47.2)	36 (7.3)	
Religion	Catholic	157 (47.6)	173 (52.4)	330 (66.8)	0.108
	Christianity	41 (59.4)	28 (40.6)	69 (14.0)	
	Atheist	21 (61.8)	13 (38.2)	34 (6.9)	
	Agnostic	16 (41.0)	23 (59.0)	39 (7.9)	
	Others	16 (72.7)	6 (27.3)	22 (4.5)	
City	Lima	228 (49.7)	231 (50.3)	459 (92.9)	0.002
	Province	23 (65.7)	12 (34.3)	35 (7.1)	
Total		251 (50.8)	243 (49.2)	494 (100)	0.049

Data in N(%).

The results on stress reveal that 31.6% of university students in Peru agree they would feel stressed if a war were to start, while 27.1% feel stressed about their inability to control the situation. 30% are concerned about the influence of international organizations and media, and 33% experience stress when hearing about the suffering of people in conflict zones. Furthermore, 31% indicated they would not know how to manage their personal problems in such a context, while 30% felt stressed by media coverage, and another 31% expressed an inability to handle personal challenges during this time (Supplementary Table 1).

### 3.2 Fear of war

Fear of war was reported by 49% (242/494) of participants, with a higher prevalence among women (53.5%, 154/288, *p* = 0.012). *Post-hoc* analysis revealed significant differences in war-related fear between men and women (*p* = 0.001). By age group, fear was most common in participants aged 51–60 years (61.5%), followed by 31–40 years (52.6%) and 21–30 years (51.0%). Fear affected 60% of divorced participants and 50.2% of singles (*p* = 0.139). Among religious affiliations, 54.5% of those identifying with other unlisted religions reported fear, followed by 51.3% agnostics and 49.1% Catholics (*p* = 0.201). Geographically, fear was more prevalent among students in Lima (50.8%) compared to those from other provinces (25.7%, *p* = 0.007; Table 3).

**Table 3:** Distribution of fear of war in Peruvian university students.

Variables	Categories	Low fear	High fear	Total	*p*-value
Sex	Male	116 (57.7)	85 (42.3)	201 (40.7)	0.012
	Female	134 (46.5)	154 (53.5)	288 (58.3)	
	No binary	2 (40)	3 (60)	5 (1.0)	
Age group (years)	18–20	56 (52.8)	50 (47.2)	106 (21.5)	0.059
	21–30	144 (49.0)	150 (51.0)	294 (59.5)	
	31–40	27 (47.4)	30 (52.6)	57 (11.5)	
	41–50	20 (83.3)	4 (16.7)	24 (4.9)	
	51–60	5 (34.5)	8 (61.5)	13 (2.6)	
Marital status	Single	206 (49.8)	208 (50.2)	414 (83.8)	0.139
	Married	25 (64.1)	14 (35.9)	39 (7.9)	
	Divorced	2 (40)	3 (60)	5 (1.0)	
	Cohabiting relationships	19 (52.8)	17 (47.2)	36 (7.3)	
Religion	Catholic	168 (50.9)	162 (49.1)	330 (66.8)	0.201
	Christianity	36 (52.2)	33 (47.8)	69 (14.0)	
	Atheist	19 (55.9)	15 (44.1)	34 (6.9)	
	Agnostic	19 (48.7)	20 (51.3)	39 (7.9)	
	Others	10 (45.5)	12 (54.5)	22 (4.5)	
Origin	Lima	226 (49.2)	233 (50.8)	459 (92.9)	0.007
	Province	26 (74.3)	9 (25.7)	35 (7.1)	
Total		252 (51.0)	242 (49.0)	494 (100)	0.044

Data in N(%).

Regarding fear, 28.7% of students reported being afraid of losing their lives due to war, and 29.1% had trouble sleeping due to worry. A significant 36.8% feared a potential food shortage, while 36.4% were concerned about disruptions in the import and export of essential goods. Additional fears included the potential use of nuclear or biological weapons (21.9%) and the possibility that family or friends near the conflict zone would be affected (30.4%; Supplementary Table 2).

### 3.3 Anxiety

Anxiety symptoms were present in 34.8% (172/494) of university students as mild anxiety, followed by 10.3% (51/494) and 2.6% (13/494) with moderate and severe anxiety, respectively (Table 4). Women experienced higher levels of anxiety overall (37.8% mild, 13.2% moderate, and 4.2% severe, *p* < 0.001). *Post-hoc* analysis shows significant differences between women and men (*p* = 0.003). Mild anxiety was most prevalent in the 18–20 age group (44.3%), followed by the 31–40 age group (38.6%) and the 51–60 age group (38.5%; *p* = 0.012). In the *post-hoc* analysis, significant differences were observed between age groups: 18–20 years versus 41–50 years (*p* = 0.021) and 51–60 years (*p* = 0.006); 21–30 years versus 41–50 years (*p* = 0.002); and 31–40 years versus 51–60 years (*p* = 0.016). Severe anxiety was less frequent, with 7.5% in the 18–20 age group and 1.7% in the 21–30 age group (*p* = 0.002).

**Table 4:** Anxiety symptoms in Peruvian university students.

Variables	Categories	Anxiety				Total	*p*-value
		No anxiety	Mild	Moderate	Severe		
Sex	Male	127 (63.2)	60 (29.9)	13 (6.5)	1 (0.5)	201 (40.7)	<0.001
	Female	129 (44.8)	109 (37.8)	38 (13.2)	12 (4.2)	288 (58.3)	
	No binary	2 (40)	3 (60)	0 (0)	0 (0)	5 (1.0)	
Age group (years)	18–20	33 (31.1)	47 (44.3)	18 (17.0)	8 (7.5)	106 (21.5)	0.002
	21–30	169 (57.5)	94 (32.0)	26 (8.8)	5 (1.7)	294 (59.5)	
	31–40	32 (56.1)	22 (38.6)	3 (5.3)	0 (0)	57 (11.5)	
	41–50	18 (75)	4 (16.7)	2 (8.3)	0 (0)	24 (4.9)	
	51–60	6 (46.2)	5 (38.5)	2 (15.4)	0 (0)	13 (2.6)	
Marital status	Single	212 (51.2)	143 (34.5)	47 (11.4)	12 (2.9)	414 (83.8)	0.009
	Married	25 (64.1)	9 (23.1)	4 (10.3)	1 (2.6)	39 (7.9)	
	Divorced	0 (0)	5 (100)	0 (0)	0 (0)	5 (1.0)	
	Cohabiting relationships	21 (58.3)	15 (41.7)	0 (0)	0 (0)	36 (7.3)	
Religion	Catholic	167 (50.6)	119 (36.1)	33 (10)	11 (3.3)	330 (66.8)	0.002
	Christianity	38 (55.1)	25 (36.2)	6 (8.7)	0 (0)	69 (14.0)	
	Atheist	18 (52.9)	10 (29.4)	5 (14.7)	1 (2.9)	34 (6.9)	
	Agnostic	20 (51.3)	16 (41.0)	2 (5.1)	1 (2.6)	39 (7.9)	
	Others	15 (68.2)	2 (9.1)	5 (22.7)	0 (0)	22 (4.5)	
Origin	Lima	241 (52.5)	157 (34.2)	48 (10.5)	13 (2.8)	459 (92.9)	0.044
	Province	17 (48.6)	15 (42.9)	3 (8.6)	0 (0)	35 (7.1)	
Total		258 (52.2)	172 (34.8)	51 (10.3)	13 (2.6)	494 (100)	0.041

Data in N(%).

All divorced participants experienced mild anxiety, followed by 41.7% of those in cohabiting relationships, 34.5% of singles, and 23.1% of married individuals (*p* = 0.009). Severe anxiety was observed in 2.9% of singles and 2.6% of married individuals (*p* = 0.323). Anxiety symptoms differed significantly based on relationship status, with higher levels in students who were single compared to those cohabiting (*p* = 0.002) or married (*p* = 0.003). Among agnostics, 41% reported mild anxiety, while 22.7% of those affiliated with other unlisted religions and 14.7% of atheists experienced moderate anxiety (*p* = 0.002). Severe anxiety was reported in 3.3% of Catholics. Analysis by religious affiliation revealed significant differences in anxiety symptoms between Catholics and Christians (*p* = 0.003), Agnostics (*p* = 0.011), and those identifying as “Other” (*p* = 0.001). Additionally, Atheists differed significantly from Agnostics (*p* = 0.021). Geographically, 42.9% of provincial students had mild anxiety, with none reporting severe anxiety, compared to 10.5% of Lima students reporting moderate anxiety and 2.8% reporting severe anxiety (*p* = 0.044).

### 3.4 Depression

Depression symptoms affected 34.8% (172/494) of participants with mild depression (Table 4). Also, we reported 10.3% (51/494), 2.4% (12/494), and 0.2% (1/494) with moderate, moderate–severe and severe depression, respectively. Women showed the highest prevalence across all categories (37.8% mild, 13.2% moderate, 3.8% moderately severe), and were the only group with a case of severe depression (1/288, 0.3%; *p* = 0.002). No significant differences in depression symptoms were found between women and non-binary individuals (*p* = 0.061), but significant differences were observed between men and non-binary individuals (*p* = 0.002). The 18–20 age group had the highest prevalence of mild (44.3%), moderate (17.0%), and moderately severe depression (7.5%; *p* = 0.007). However, only 0.3% (1/294) of participants aged 21–30 years experienced severe depression (*p* = 0.001). In the *post-hoc* analysis, significant differences in depression symptoms were observed between age groups: 21–30 years versus 41–50 years (*p* = 0.007) and 51–60 years (*p* = 0.037); 18–20 years versus 41–50 years (*p* = 0.003) and 51–60 years (*p* = 0.041); and 31–40 years versus 51–60 years (*p* = 0.002).

Among marital statuses, 41.7% of those in cohabiting relationships experienced mild depression, followed by 34.5% of singles, who also had the highest frequency of moderate depression (11.4%), moderately severe depression (2.7%), and severe depression (0.2%; *p* = 0.022). Depression symptoms also varied by relationship status, with significant differences between single and cohabiting students (*p* = 0.022), married and cohabiting students (*p* = 0.011), and single and married students (*p* = 0.005). Catholics were the only group reporting all levels of depression severity (36.1% mild, 10% moderate, 3% moderately severe, and 0.3% severe; *p* = 0.009). *Post-hoc* analysis by religious affiliation revealed significant differences in depression symptoms between Catholics and atheists (*p* = 0.002), agnostics (*p* = 0.008), and those identifying as “Other” (*p* = 0.036), as well as between atheists and other religious groups (*p* = 0.004). Finally, provincial students exhibited higher rates of mild depression (42.5%) and moderately severe depression (8.6%), while among Lima students, 10.5% reported moderate depression, 2.6% moderately severe depression, and 0.2% severe depression (*p* = 0.002; Table 5).

**Table 5:** Depression symptoms in Peruvian university students.

Variables	Categories	Depression					Total	*p*-value
		No depression	Mild	Moderate	Moderately severe	Severe		
Sex	Male	127 (63.2)	60 (29.9)	13 (6.5)	1 (0.5)	0 (0)	201 (40.7)	0.002
	Female	129 (44.8)	109 (37.8)	38 (13.2)	11 (3.8)	1 (0.3)	288 (58.3)	
	No binary	2 (40)	3 (60)	0 (0)	0 (0)	0 (0)	5 (1.0)	
Age group (years)	18–20	33 (31.1)	47 (44.3)	18 (17.0)	8 (7.5)	0 (0)	106 (21.5)	0.038
	21–30	169 (57.5)	94 (32.0)	26 (8.8)	4 (1.4)	1 (0.3)	294 (59.5)	
	31–40	32 (56.1)	22 (38.6)	3 (5.3)	0 (0)	0 (0)	57 (11.5)	
	41–50	18 (75)	4 (16.7)	2 (8.3)	0 (0)	0 (0)	24 (4.9)	
	51–60	6 (46.2)	5 (38.5)	2 (15.4)	0 (0)	0 (0)	13 (2.6)	
Marital status	Single	212 (51.2)	143 (34.5)	47 (11.4)	11 (2.7)	1 (0.2)	414 (83.8)	0.022
	Married	25 (64.1)	9 (23.1)	4 (10.3)	1 (2.6)	0 (0)	39 (7.9)	
	Divorced	0 (0)	5 (100)	0 (0)	0 (0)	0 (0)	5 (1.0)	
	Cohabiting relationships	21 (58.3)	15 (41.7)	0 (0)	0 (0)	0 (0)	36 (7.3)	
Religion	Catholic	167 (50.6)	119 (36.1)	33 (10)	10 (3.0)	1 (0.3)	330 (66.8)	0.009
	Christianity	38 (55.1)	25 (36.2)	6 (8.7)	0 (0)	0 (0)	69 (14.0)	
	Atheist	18 (52.9)	10 (29.4)	5 (14.7)	1 (2.9)	0 (0)	34 (6.9)	
	Agnostic	20 (51.3)	16 (41.0)	2 (5.1)	1 (2.6)	0 (0)	39 (7.9)	
	Others	15 (68.2)	2 (9.1)	5 (22.7)	0 (0)	0 (0)	22 (4.5)	
City	Lima	241 (52.5)	157 (34.2)	48 (10.5)	12 (2.6)	1 (0.2)	459 (92.9)	0.002
	Province	17 (48.6)	15 (42.9)	3 (8.6)	0 (0)	0 (0)	35 (7.1)	
Total		258 (52.2)	172 (34.8)	51 (10.3)	12 (2.4)	1 (0.2)	494 (100)	0.033

Data in N(%).

## 4 Discussion

This study reveals that stress and fear are prevalent emotions among university students in response to the imminent possibility of war. Nearly half of the Peruvian students reported experiencing mental discomfort due to the Russia-Ukraine conflict, with women being disproportionately affected. Stress and fear were more common among divorced individuals aged 51–60, and residents of Lima, while mild anxiety and depression were more frequent in younger students aged 18–20 and those living in provinces.

### 4.1 Strengths

To the best of our knowledge, this is the first study to evaluate anxiety, depression, fear of war, and stress among Latin American university students during the onset of the Russia-Ukraine conflict. Other studies tend to focus on the general population and often overlook South American countries in their analyses (15, 34, 35). Furthermore, several investigations fail to assess the specific symptom of war-related fear (34, 36), which can significantly influence the severity of other mental health disorders (15). Our research contributes important scientific insights for Spanish-speaking countries, particularly as the university student population remains largely understudied (36, 37).

### 4.2 Main discussion

The Russia-Ukraine conflict has negatively impacted populations worldwide (9, 10). Understandably, the Ukrainian population directly affected by the war experiences high levels of depression, anxiety, and stress. However, the conflict has also significantly affected neighboring countries like Poland and Romania, with 80.3% of Poles and 47.1% of Romanians fearing a potential Russian invasion—both influenced by war images disseminated in the media (34, 35).

This phenomenon of anxiety and fear is not limited to neighboring countries. Even in distant regions such as East Asia (Taiwan), rising concerns about the conflict have been observed (34). Social media and real-time conventional media coverage play a crucial role in creating a “virtual proximity” to the war, amplifying fear worldwide (38). This pattern is also evident in Latin America, despite the geographical distance, where strong associations have been found between the fear of global armed conflict and severe levels of anxiety, depression, or stress (15). In these regions, the conflict exacerbates preexisting political and socioeconomic challenges, worsened by the COVID-19 pandemic, which has deeply impacted mental health (15). This may explain why nearly half of the students in our sample reported symptoms of anxiety and depression despite not being directly involved in the conflict. Additionally, the third group of participants expressed fear of a potential international economic blockade, stress upon learning about the suffering of people in war-affected countries, and concern about food and essential product shortages.

Multiple studies have demonstrated that younger populations and women are more likely to experience elevated levels of stress, anxiety, and depression during the Russia-Ukraine conflict (15). This trend is particularly concerning in Peru, where most university students are between 18 and 25 years old, and 51% are women (23). Our findings reflect this reality, with mild anxiety and depression affecting nearly a third of students, especially those aged 18–20. More than half of the women reported high levels of stress and fear, along with a greater prevalence of anxiety and depression symptoms across various categories.

These findings suggest that university students, unlike other populations, are particularly vulnerable to the psychological impact of armed conflicts. This conclusion aligns with a study conducted in four Ukrainian universities, which found that fear, stress, and depression more frequently affected students compared to university staff, with women being the most affected (37). Researchers attribute these results to the disruption of daily routines and loss of connection with family and friends. In our study, about one-third of students also expressed fear of being unable to continue their studies, work, or daily tasks, reflecting a similar concern to that of Ukrainian students. Moreover, the COVID-19 pandemic has drastically altered the study dynamics and daily routines of Peruvian students (39–41), potentially worsening mental health issues caused by the 2022 conflict.

A systematic review (36) that included university students from various continents (America, Asia, Europe, etc.) supports the findings of this research, reporting stress rates ranging from 28.14 to 56% and anxiety rates from 13.63 to 88.9%. In addition to the factors mentioned, other predictors of these disorders were identified, such as loneliness, poor academic performance, urban confinement, family or friends contracting COVID-19, graduation delays, canceled planned events, and the inability to meet pre-crisis financial goals (36). These data are especially concerning given the low resilience observed in university students, indicating a reduced capacity to cope with adversity (37). This underscores the need for significant efforts to help young people manage the mental health consequences not only of armed conflict but also the prolonged impact of the COVID-19 pandemic.

### 4.3 Limitations

Although this was not a single-center study, most participants were concentrated in Peru’s capital, which may limit the generalizability of the findings. Future research should include more diverse populations, particularly from regions outside the capital, such as the jungle and highland areas of Peru, to identify contextual factors that may influence the development of mental health disorders (36, 42). Cultural, socioeconomic, and resource access differences in these areas could play a significant role in the onset of anxiety, depression, and stress.

Additionally, key factors related to professional development, such as academic level or delayed graduation, were not considered, although they may have a substantial impact on the emergence of these disorders (43). Likewise, preexisting medical conditions or having family members or friends infected with COVID-19 were not evaluated, yet these have been shown to be important predictors of deteriorating mental health in other studies (36).

Another limitation was the absence of specific subgroup analyses, such as students with a history of mental disorders, substance use, or post-COVID sequelae. Further research should include such analyses to assess the potential synergistic effects of these factors in combination with other crises, such as armed conflicts or future pandemics. Finally, self-selection bias due to web-based data collection could be another limitation of this study. Despite these limitations, this study is one of the first to demonstrate the effects of the Russia-Ukraine conflict in Peru.

## 5 Conclusion

This study demonstrates the significant impact of the imminent armed conflict on the mental health of Peruvian university students, with high levels of stress and fear. University students have experienced considerable psychological distress due to the Russia-Ukraine conflict, exhibiting symptoms of stress, fear, anxiety, and depression, despite not being directly involved. Women and younger students were identified as the most vulnerable groups to these disorders.

War-related stress and fear have a profound psychological impact on this student population, raising concerns about their emotional well-being in times of global uncertainty. These findings highlight the far-reaching psychological effects of global conflicts, even on populations geographically distant from the war zone, emphasizing the emotional burden experienced during international crises. Further research should include longitudinal studies to assess changes over time or qualitative approaches to explore personal experiences more deeply during emergency contexts.

## Data Availability

The original contributions presented in the study are included in the article/Supplementary material, further inquiries can be directed to the corresponding authors.
