# Supplementary material for: Peruvian university students’ mental health in crisis: assessing anxiety, depression, fear, and stress during the Russia-Ukraine conflict
Source: Front Public Health. 2025 Mar 27;13:1522132. doi: 10.3389/fpubh.2025.1522132 (PMC11983601; doi:10.3389/fpubh.2025.1522132)
Supplement: Supplementary file 1 [file Table_1.DOCX]

**Suppl. 1** Characteristics related to the Russia-Ukraine conflict that generate stress symptoms. Data in N(%)

| **Stress** | | | | | |
| --- | --- | --- | --- | --- | --- |
| In this context of imminent/very possible war, I am stressed by: | **Strongly disagree** | **Disagree** | **Indifferent** | **Agree** | **Strongly agree** |
| Feeling that I will be affected by war, if it starts | 132 (26.7) | 111 (22.5) | 87 (17.6) | 156 (31.6) | 8 (1.6) |
| Being unable to control what happens in the context of war | 134 (27.1) | 112 (22.7) | 98 (19.8) | 134 (27.1) | 16 (3.2) |
| Not knowing how to handle my problems in the face of war | 153 (31.0) | 137 (27.7) | 109 (22.1) | 81 (16.4) | 14 (2.8) |
| The decisions made by politicians | 130 (26.3) | 110 (22.3) | 78 (15.8) | 131 (26.5) | 45 (9.1) |
| The decisions made by international organizations (UN, NATO, etc.) | 125 (25.3) | 100 (20.2) | 77 (15.6) | 148 (30.0) | 44 (8.9) |
| The situation is shown to us by the media | 122 (24.7) | 112 (22.7) | 86 (17.4) | 146 (29.6) | 28 (5.7) |
| This situation gets out of control and a bigger war breaks out | 127 (25.7) | 103 (20.9) | 63 (12.8) | 148 (30.0) | 43 (8.7) |
| To know the number of dead and/or wounded | 125 (25.3) | 99 (20.0) | 89 (18.0) | 146 (29.6) | 35 (7.1) |
| To know about the suffering of the people who live in the countries involved | 122 (24.7) | 92 (18.6) | 68 (13.8) | 163 (33.0) | 49 (9.9) |
| The damage to the environment that war can cause | 117 (23.7) | 96 (19.4) | 56 (11.3) | 149 (30.2) | 76 (15.4) |

**Suppl. 2** Characteristics related to the Russia-Ukraine conflict that generate fear of war. Data in N(%)

| **Fear of war** | | | | | |
| --- | --- | --- | --- | --- | --- |
| In the face of an imminent/very possible world war, I am so afraid or worried: | **Strongly disagree** | **Disagree** | **Indifferent** | **Agree** | **Strongly agree** |
| I am afraid of losing my life because of the war | 142 (28.7) | 119 (24.1) | 86 (17.4) | 116 (23.5) | 31 (6.3) |
| I am not able to sleep | 143 (28.9) | 144 (29.1) | 118 (23.9) | 74 (15.0) | 15 (3.0) |
| My heart races or palpitates when I think about a new world war | 153 (31.0) | 149 (30.2) | 95 (19.2) | 83 (16.8) | 14 (2.8) |
| That soldiers from my country are asked to go to war | 134 (27.1) | 122 (24.7) | 86 (17.4) | 129 (26.1) | 23 (4.7) |
| My political leaders ask us to join the armed conflict as a country | 136 (27.5) | 132 (26.7) | 72 (14.6) | 113 (22.9) | 41 (8.3) |
| That atomic/nuclear, biological, or mass destruction bombs are launched or used on us | 146 (29.6) | 100 (20.2) | 73 (14.8) | 108 (21.9) | 67 (13.6) |
| I am afraid or worried that this war will cause food shortages | 92 (18.6) | 64 (13.0) | 30 (6.1) | 182 (36.8) | 126 (25.5) |
| We will not be able to import/export resources from the countries in conflict | 83 (16.8) | 77 (15.6) | 50 (10.1) | 180 (36.4) | 104 (21.1) |
| Other essential resources will become scarce due to the war | 92 (18.6) | 59 (11.9) | 36 (7.3) | 182 (36.8) | 125 (25.3) |
| All types of financial transactions with other countries will be blocked | 91 (18.4) | 76 (15.4) | 55 (11.1) | 185 (37.4) | 87 (17.6) |
| Properties (houses, premises, etc.) will be devalued | 103 (20.9) | 75 (15.2) | 76 (15.4) | 157 (31.8) | 83 (16.8) |
| The stock market will collapse | 97 (19.6) | 72 (14.6) | 58 (11.7) | 168 (34.0) | 99 (20.4) |
| Oil and other fuel prices rise due to war | 93 (18.8) | 64 (13.0) | 46 (9.3) | 178 (36.0) | 113 (22.9) |
| My family and friends who live near the war zone are affected | 113 (22.9) | 67 (13.6) | 63 (12.8) | 150 (30.4) | 101 (20.4) |
| They restrict our exit or free movement | 102 (20.4) | 71 (14.4) | 64 (13.0) | 162 (32.8) | 95 (19.3) |
| I cannot study, work or do my daily duties | 104 (21.1) | 69 (14.0) | 52 (10.5) | 155 (31.4) | 144 (29.1) |
